# Supplementary material for: Heparin-based hydrogel scaffolding alters the transcriptomic profile and increases the chemoresistance of MDA-MB-231 triple-negative breast cancer cells
Source: Biomater Sci. 2020 Feb 13;8(10):2786–96. doi: 10.1039/c9bm01481k (PMC7497406; doi:10.1039/c9bm01481k)
Supplement: Supplementary file 2 [file BM-008-C9BM01481K-s002.zip › Supplementary File 4/HP-BhydrogelvControl/Pathways/my_analysis.Gsea.1545201079907/HALLMARK_MYC_TARGETS_V1.html]

Details for gene set HALLMARK\_MYC\_TARGETS\_V1[GSEA]

|  || Dataset | expr.class.cls#HYD\_versus\_CONTROL.class.cls#HYD\_versus\_CONTROL\_repos |
| Phenotype | class.cls#HYD\_versus\_CONTROL\_repos |
| Upregulated in class | CONTROL |
| GeneSet | HALLMARK\_MYC\_TARGETS\_V1 |
| Enrichment Score (ES) | -0.55535704 |
| Normalized Enrichment Score (NES) | -2.6377854 |
| Nominal p-value | 0.0 |
| FDR q-value | 0.0 |
| FWER p-Value | 0.0 |
Table: GSEA Results Summary

  

Fig 1: Enrichment plot: HALLMARK\_MYC\_TARGETS\_V1      
 Profile of the Running ES Score & Positions of GeneSet Members on the Rank Ordered List

  

| PROBE | DESCRIPTION (from dataset) | GENE SYMBOL | GENE\_TITLE | RANK IN GENE LIST | RANK METRIC SCORE | RUNNING ES | CORE ENRICHMENT || 1 | EIF4A1 | na |  |  | 1639 | 1.516 | -0.0755 | No |
| 2 | RRP9 | na |  |  | 1722 | 1.481 | -0.0694 | No |
| 3 | PRPF31 | na |  |  | 2727 | 1.109 | -0.1143 | No |
| 4 | EIF3B | na |  |  | 3135 | 1.002 | -0.1287 | No |
| 5 | TRIM28 | na |  |  | 3236 | 0.980 | -0.1270 | No |
| 6 | RNPS1 | na |  |  | 3652 | 0.881 | -0.1426 | No |
| 7 | MYC | na |  |  | 3771 | 0.854 | -0.1428 | No |
| 8 | CAD | na |  |  | 4030 | 0.806 | -0.1507 | No |
| 9 | NOP16 | na |  |  | 4286 | 0.755 | -0.1587 | No |
| 10 | PABPC4 | na |  |  | 4583 | 0.695 | -0.1694 | No |
| 11 | NOP56 | na |  |  | 4859 | 0.647 | -0.1793 | No |
| 12 | TRA2B | na |  |  | 4957 | 0.634 | -0.1799 | No |
| 13 | MCM7 | na |  |  | 5533 | 0.548 | -0.2063 | No |
| 14 | MCM2 | na |  |  | 5595 | 0.539 | -0.2057 | No |
| 15 | RUVBL2 | na |  |  | 5708 | 0.524 | -0.2079 | No |
| 16 | EXOSC7 | na |  |  | 5715 | 0.523 | -0.2045 | No |
| 17 | SRM | na |  |  | 6386 | 0.433 | -0.2367 | No |
| 18 | U2AF1 | na |  |  | 6417 | 0.427 | -0.2352 | No |
| 19 | CDC45 | na |  |  | 6492 | 0.417 | -0.2362 | No |
| 20 | IFRD1 | na |  |  | 7053 | 0.353 | -0.2631 | No |
| 21 | PSMC4 | na |  |  | 7347 | 0.321 | -0.2763 | No |
| 22 | TFDP1 | na |  |  | 7738 | 0.282 | -0.2948 | No |
| 23 | MCM5 | na |  |  | 8383 | 0.209 | -0.3272 | No |
| 24 | CYC1 | na |  |  | 8715 | 0.179 | -0.3434 | No |
| 25 | MCM4 | na |  |  | 8791 | 0.168 | -0.3461 | No |
| 26 | HDGF | na |  |  | 8798 | 0.167 | -0.3453 | No |
| 27 | ODC1 | na |  |  | 8852 | 0.159 | -0.3469 | No |
| 28 | NOLC1 | na |  |  | 9142 | 0.135 | -0.3612 | No |
| 29 | AIMP2 | na |  |  | 9143 | 0.135 | -0.3602 | No |
| 30 | RANBP1 | na |  |  | 9418 | 0.103 | -0.3739 | No |
| 31 | POLD2 | na |  |  | 9616 | 0.087 | -0.3837 | No |
| 32 | CDC20 | na |  |  | 9617 | 0.086 | -0.3831 | No |
| 33 | EIF3D | na |  |  | 9654 | 0.079 | -0.3844 | No |
| 34 | CSTF2 | na |  |  | 9693 | 0.076 | -0.3858 | No |
| 35 | TYMS | na |  |  | 9817 | 0.062 | -0.3919 | No |
| 36 | FAM120A | na |  |  | 9845 | 0.059 | -0.3929 | No |
| 37 | PPM1G | na |  |  | 10505 | -0.006 | -0.4275 | No |
| 38 | HNRNPU | na |  |  | 10586 | -0.015 | -0.4316 | No |
| 39 | VBP1 | na |  |  | 10702 | -0.030 | -0.4374 | No |
| 40 | SRSF3 | na |  |  | 10721 | -0.032 | -0.4382 | No |
| 41 | SF3A1 | na |  |  | 10926 | -0.055 | -0.4485 | No |
| 42 | TXNL4A | na |  |  | 10985 | -0.064 | -0.4511 | No |
| 43 | MAD2L1 | na |  |  | 11353 | -0.097 | -0.4697 | No |
| 44 | SRSF1 | na |  |  | 11410 | -0.102 | -0.4720 | No |
| 45 | DDX21 | na |  |  | 11428 | -0.105 | -0.4721 | No |
| 46 | NME1 | na |  |  | 11542 | -0.117 | -0.4772 | No |
| 47 | MCM6 | na |  |  | 11728 | -0.142 | -0.4860 | No |
| 48 | RPS2 | na |  |  | 11795 | -0.149 | -0.4884 | No |
| 49 | PA2G4 | na |  |  | 11816 | -0.152 | -0.4884 | No |
| 50 | SNRPD1 | na |  |  | 11926 | -0.166 | -0.4929 | No |
| 51 | SF3B3 | na |  |  | 11935 | -0.168 | -0.4922 | No |
| 52 | PSMA7 | na |  |  | 11991 | -0.172 | -0.4938 | No |
| 53 | DEK | na |  |  | 12016 | -0.175 | -0.4939 | No |
| 54 | NCBP1 | na |  |  | 12144 | -0.192 | -0.4992 | No |
| 55 | KARS | na |  |  | 12158 | -0.193 | -0.4985 | No |
| 56 | PCBP1 | na |  |  | 12214 | -0.201 | -0.5000 | No |
| 57 | CCT5 | na |  |  | 12253 | -0.207 | -0.5005 | No |
| 58 | SET | na |  |  | 12332 | -0.218 | -0.5031 | No |
| 59 | SMARCC1 | na |  |  | 12750 | -0.252 | -0.5232 | No |
| 60 | UBA2 | na |  |  | 12764 | -0.254 | -0.5221 | No |
| 61 | RAD23B | na |  |  | 12787 | -0.256 | -0.5215 | No |
| 62 | CBX3 | na |  |  | 12874 | -0.267 | -0.5241 | No |
| 63 | GOT2 | na |  |  | 12892 | -0.271 | -0.5231 | No |
| 64 | SERBP1 | na |  |  | 12966 | -0.279 | -0.5249 | No |
| 65 | SNRPA1 | na |  |  | 13056 | -0.292 | -0.5276 | No |
| 66 | TUFM | na |  |  | 13260 | -0.317 | -0.5360 | No |
| 67 | SRSF7 | na |  |  | 13290 | -0.323 | -0.5352 | No |
| 68 | CCT2 | na |  |  | 13310 | -0.327 | -0.5339 | No |
| 69 | ORC2 | na |  |  | 13360 | -0.330 | -0.5342 | No |
| 70 | TARDBP | na |  |  | 13387 | -0.335 | -0.5332 | No |
| 71 | CLNS1A | na |  |  | 13522 | -0.356 | -0.5377 | No |
| 72 | DDX18 | na |  |  | 13554 | -0.361 | -0.5368 | No |
| 73 | RAN | na |  |  | 13684 | -0.383 | -0.5409 | No |
| 74 | C1QBP | na |  |  | 13784 | -0.400 | -0.5432 | No |
| 75 | BUB3 | na |  |  | 13790 | -0.401 | -0.5407 | No |
| 76 | IARS | na |  |  | 13999 | -0.416 | -0.5487 | No |
| 77 | RFC4 | na |  |  | 14127 | -0.430 | -0.5523 | Yes |
| 78 | STARD7 | na |  |  | 14133 | -0.431 | -0.5495 | Yes |
| 79 | DHX15 | na |  |  | 14160 | -0.435 | -0.5478 | Yes |
| 80 | ABCE1 | na |  |  | 14186 | -0.439 | -0.5460 | Yes |
| 81 | PRPS2 | na |  |  | 14222 | -0.446 | -0.5447 | Yes |
| 82 | VDAC3 | na |  |  | 14267 | -0.455 | -0.5438 | Yes |
| 83 | PSMC6 | na |  |  | 14342 | -0.469 | -0.5444 | Yes |
| 84 | HSPE1 | na |  |  | 14343 | -0.470 | -0.5411 | Yes |
| 85 | PSMA2 | na |  |  | 14390 | -0.475 | -0.5401 | Yes |
| 86 | COPS5 | na |  |  | 14404 | -0.477 | -0.5375 | Yes |
| 87 | ERH | na |  |  | 14453 | -0.483 | -0.5366 | Yes |
| 88 | SLC25A3 | na |  |  | 14602 | -0.506 | -0.5408 | Yes |
| 89 | G3BP1 | na |  |  | 14666 | -0.518 | -0.5404 | Yes |
| 90 | KPNB1 | na |  |  | 14673 | -0.519 | -0.5371 | Yes |
| 91 | NHP2 | na |  |  | 14692 | -0.521 | -0.5343 | Yes |
| 92 | CUL1 | na |  |  | 14693 | -0.522 | -0.5306 | Yes |
| 93 | PSMD3 | na |  |  | 14709 | -0.525 | -0.5277 | Yes |
| 94 | USP1 | na |  |  | 14776 | -0.533 | -0.5274 | Yes |
| 95 | HSPD1 | na |  |  | 14793 | -0.536 | -0.5245 | Yes |
| 96 | CCNA2 | na |  |  | 14794 | -0.537 | -0.5207 | Yes |
| 97 | CNBP | na |  |  | 14843 | -0.543 | -0.5194 | Yes |
| 98 | MRPL9 | na |  |  | 14883 | -0.546 | -0.5176 | Yes |
| 99 | GNL3 | na |  |  | 14904 | -0.551 | -0.5147 | Yes |
| 100 | PTGES3 | na |  |  | 14997 | -0.561 | -0.5156 | Yes |
| 101 | PSMD14 | na |  |  | 15161 | -0.591 | -0.5200 | Yes |
| 102 | SNRPG | na |  |  | 15196 | -0.594 | -0.5176 | Yes |
| 103 | FBL | na |  |  | 15209 | -0.597 | -0.5140 | Yes |
| 104 | SSB | na |  |  | 15232 | -0.602 | -0.5109 | Yes |
| 105 | XPOT | na |  |  | 15237 | -0.603 | -0.5069 | Yes |
| 106 | EIF1AX | na |  |  | 15275 | -0.612 | -0.5045 | Yes |
| 107 | GSPT1 | na |  |  | 15339 | -0.624 | -0.5034 | Yes |
| 108 | PSMA4 | na |  |  | 15398 | -0.634 | -0.5020 | Yes |
| 109 | SNRPD2 | na |  |  | 15424 | -0.637 | -0.4988 | Yes |
| 110 | PSMA6 | na |  |  | 15498 | -0.650 | -0.4980 | Yes |
| 111 | RRM1 | na |  |  | 15628 | -0.673 | -0.5001 | Yes |
| 112 | ETF1 | na |  |  | 15634 | -0.675 | -0.4955 | Yes |
| 113 | POLE3 | na |  |  | 15652 | -0.677 | -0.4917 | Yes |
| 114 | PHB2 | na |  |  | 15698 | -0.681 | -0.4892 | Yes |
| 115 | H2AFZ | na |  |  | 15730 | -0.688 | -0.4860 | Yes |
| 116 | CCT3 | na |  |  | 15867 | -0.718 | -0.4881 | Yes |
| 117 | EIF4H | na |  |  | 15869 | -0.718 | -0.4830 | Yes |
| 118 | MRPL23 | na |  |  | 15878 | -0.720 | -0.4784 | Yes |
| 119 | RPL18 | na |  |  | 15882 | -0.720 | -0.4735 | Yes |
| 120 | PCNA | na |  |  | 15928 | -0.728 | -0.4707 | Yes |
| 121 | UBE2L3 | na |  |  | 15929 | -0.728 | -0.4655 | Yes |
| 122 | XRCC6 | na |  |  | 15966 | -0.737 | -0.4622 | Yes |
| 123 | HDDC2 | na |  |  | 16079 | -0.766 | -0.4627 | Yes |
| 124 | EIF2S1 | na |  |  | 16087 | -0.767 | -0.4577 | Yes |
| 125 | EIF2S2 | na |  |  | 16160 | -0.780 | -0.4559 | Yes |
| 126 | PHB | na |  |  | 16365 | -0.825 | -0.4608 | Yes |
| 127 | IMPDH2 | na |  |  | 16371 | -0.826 | -0.4553 | Yes |
| 128 | NCBP2 | na |  |  | 16490 | -0.851 | -0.4555 | Yes |
| 129 | SRSF2 | na |  |  | 16515 | -0.858 | -0.4507 | Yes |
| 130 | GLO1 | na |  |  | 16520 | -0.859 | -0.4448 | Yes |
| 131 | RPS3 | na |  |  | 16543 | -0.865 | -0.4398 | Yes |
| 132 | HNRNPA2B1 | na |  |  | 16592 | -0.870 | -0.4362 | Yes |
| 133 | CCT7 | na |  |  | 16606 | -0.873 | -0.4307 | Yes |
| 134 | SNRPA | na |  |  | 16651 | -0.887 | -0.4268 | Yes |
| 135 | TCP1 | na |  |  | 16675 | -0.892 | -0.4217 | Yes |
| 136 | SSBP1 | na |  |  | 16831 | -0.935 | -0.4232 | Yes |
| 137 | SRPK1 | na |  |  | 16835 | -0.937 | -0.4168 | Yes |
| 138 | RSL1D1 | na |  |  | 16914 | -0.961 | -0.4141 | Yes |
| 139 | CDK4 | na |  |  | 16963 | -0.974 | -0.4097 | Yes |
| 140 | XPO1 | na |  |  | 16967 | -0.975 | -0.4030 | Yes |
| 141 | HDAC2 | na |  |  | 16976 | -0.979 | -0.3965 | Yes |
| 142 | SNRPB2 | na |  |  | 17013 | -0.991 | -0.3914 | Yes |
| 143 | EIF3J | na |  |  | 17206 | -1.053 | -0.3941 | Yes |
| 144 | HNRNPA1 | na |  |  | 17298 | -1.083 | -0.3912 | Yes |
| 145 | CDK2 | na |  |  | 17319 | -1.090 | -0.3845 | Yes |
| 146 | HNRNPA3 | na |  |  | 17336 | -1.097 | -0.3776 | Yes |
| 147 | KPNA2 | na |  |  | 17340 | -1.098 | -0.3700 | Yes |
| 148 | PSMB2 | na |  |  | 17433 | -1.128 | -0.3669 | Yes |
| 149 | RPL6 | na |  |  | 17483 | -1.148 | -0.3614 | Yes |
| 150 | RPL14 | na |  |  | 17510 | -1.159 | -0.3546 | Yes |
| 151 | LSM2 | na |  |  | 17527 | -1.165 | -0.3472 | Yes |
| 152 | YWHAQ | na |  |  | 17587 | -1.191 | -0.3418 | Yes |
| 153 | CCT4 | na |  |  | 17597 | -1.196 | -0.3339 | Yes |
| 154 | DUT | na |  |  | 17602 | -1.198 | -0.3256 | Yes |
| 155 | APEX1 | na |  |  | 17668 | -1.228 | -0.3204 | Yes |
| 156 | PSMD7 | na |  |  | 17688 | -1.236 | -0.3126 | Yes |
| 157 | SYNCRIP | na |  |  | 17710 | -1.247 | -0.3049 | Yes |
| 158 | NDUFAB1 | na |  |  | 17728 | -1.254 | -0.2970 | Yes |
| 159 | EIF4E | na |  |  | 17756 | -1.264 | -0.2894 | Yes |
| 160 | NAP1L1 | na |  |  | 17791 | -1.282 | -0.2822 | Yes |
| 161 | PSMD1 | na |  |  | 17797 | -1.284 | -0.2734 | Yes |
| 162 | HNRNPC | na |  |  | 17841 | -1.304 | -0.2664 | Yes |
| 163 | PRDX4 | na |  |  | 17878 | -1.323 | -0.2590 | Yes |
| 164 | LSM7 | na |  |  | 17900 | -1.334 | -0.2506 | Yes |
| 165 | SNRPD3 | na |  |  | 17968 | -1.361 | -0.2445 | Yes |
| 166 | RPS6 | na |  |  | 17988 | -1.374 | -0.2358 | Yes |
| 167 | EEF1B2 | na |  |  | 17993 | -1.377 | -0.2263 | Yes |
| 168 | RPS5 | na |  |  | 17996 | -1.380 | -0.2167 | Yes |
| 169 | HNRNPD | na |  |  | 18008 | -1.388 | -0.2074 | Yes |
| 170 | HNRNPR | na |  |  | 18147 | -1.463 | -0.2044 | Yes |
| 171 | PRDX3 | na |  |  | 18171 | -1.480 | -0.1951 | Yes |
| 172 | PWP1 | na |  |  | 18305 | -1.566 | -0.1910 | Yes |
| 173 | ILF2 | na |  |  | 18324 | -1.586 | -0.1808 | Yes |
| 174 | PSMD8 | na |  |  | 18328 | -1.587 | -0.1697 | Yes |
| 175 | PSMA1 | na |  |  | 18344 | -1.598 | -0.1592 | Yes |
| 176 | COX5A | na |  |  | 18472 | -1.687 | -0.1540 | Yes |
| 177 | YWHAE | na |  |  | 18488 | -1.699 | -0.1428 | Yes |
| 178 | ACP1 | na |  |  | 18505 | -1.708 | -0.1316 | Yes |
| 179 | RPL34 | na |  |  | 18522 | -1.723 | -0.1202 | Yes |
| 180 | MRPS18B | na |  |  | 18538 | -1.735 | -0.1087 | Yes |
| 181 | VDAC1 | na |  |  | 18615 | -1.802 | -0.1000 | Yes |
| 182 | EPRS | na |  |  | 18657 | -1.855 | -0.0891 | Yes |
| 183 | PSMB3 | na |  |  | 18785 | -1.993 | -0.0817 | Yes |
| 184 | UBE2E1 | na |  |  | 18847 | -2.083 | -0.0702 | Yes |
| 185 | PGK1 | na |  |  | 18894 | -2.154 | -0.0574 | Yes |
| 186 | HPRT1 | na |  |  | 18898 | -2.164 | -0.0422 | Yes |
| 187 | RPL22 | na |  |  | 19036 | -2.485 | -0.0319 | Yes |
| 188 | RPS10 | na |  |  | 19070 | -2.605 | -0.0152 | Yes |
| 189 | AP3S1 | na |  |  | 19148 | -3.097 | 0.0026 | Yes |
Table: GSEA details [plain text format]

  

Fig 2: HALLMARK\_MYC\_TARGETS\_V1      
 Blue-Pink O' Gram in the Space of the Analyzed GeneSet

  

Fig 3: HALLMARK\_MYC\_TARGETS\_V1: Random ES distribution      
 Gene set null distribution of ES for **HALLMARK\_MYC\_TARGETS\_V1**
